# The impacts of new quality productive forces, industrial agglomeration and energy intensity on carbon emission reduction

**DOI:** 10.1038/s41598-026-54719-2

**Published:** 2026-05-23

**Authors:** Canghong Wang

**Affiliations:** School of Accounting and Finance, Xi’an Peihua University, Xi’an, 710125 Shaanxi China

**Keywords:** New quality productive forces, Carbon emission reduction, Industrial agglomeration effect, Energy intensity, Chain mediation effect, Regional heterogeneity, Environmental sciences, Environmental social sciences, Environmental studies

## Abstract

This study investigates the impact of new quality productive forces (NQPF) on carbon emission reduction in China, with a particular focus on the mediating roles of industrial agglomeration effect (IAE) and energy intensity (EI). Using panel data covering 30 provincial-level regions in China over the period 2003–2023, we control for key confounding factors including urbanization, industrialization, industrial structure upgrading, human capital, and foreign direct investment to empirically examine the relationship between NQPF and carbon emission reduction as well as its underlying transmission mechanisms. The results indicate that NQPF exerts a significantly negative effect on carbon emissions, with this carbon reduction impact being particularly prominent in economically developed regions and eastern coastal areas of China. Further mediation analysis reveals that IAE and EI function as two critical pathways through which NQPF influences carbon emission reduction. Specifically, the enhancement of IAE reinforces the carbon reduction effect of NQPF, whereas an increase in EI weakens this effect. Overall, our findings highlight that the development of NQPF can serve as a long-term driving mechanism for effectively achieving carbon emission reduction targets, thereby providing valuable policy implications for advancing low-carbon transition and green sustainable development in China’s economy and society.

## Introduction

The ecological crisis induced by global warming has emerged as a core challenge impeding humanity’s sustainable development. Carbon emission reduction constitutes a key measure for addressing climate change. It serves as the central pathway to achieving China’s Dual Carbon goals of reaching peak carbon by 2030 and attaining carbon neutrality by 2060. It also represents an imperative requirement for advancing green economic and social transformation. Carbon reduction not only alleviates pressures from ecological degradation and mitigates the adverse impacts of climate risks on labor supply and economic activities but also drives the optimization of energy structures and industrial upgrading. This process fosters a virtuous cycle of environmental protection, innovation and growth^1^. However, as the world’s largest developing country, China faces the pressure of balancing development rights with emission reduction responsibilities. On the one hand, traditional industries’ dependence on fossil fuels imposes rigid constraints, with energy-intensive sectors still playing a pivotal role in employment security and economic growth. On the other hand, regional development imbalances lead to carbon emission reduction characterized by distinct bases, divergent potentials and uneven costs. This makes it challenging to apply uniform reduction policies across regions at different developmental stages. Against this backdrop, new quality productive forces (NQPF) represent a novel solution to the dilemma of reconciling emission reduction with growth. NQPF refer to a new form of productivity driven by technological innovation, guided by green and low-carbon principles and supported by digital transformation^2^.

Existing research on carbon emission reduction primarily progresses along three main strands. First, at the policy tool level, scholars focus on the incentive effects of environmental taxes, carbon trading markets and green finance on emission reduction. For instance, Zhang et al.^3^ found that raising tax thresholds significantly encouraged green innovation among enterprises, especially state-owned and heavily polluting enterprises in eastern regions. Second, at the technology-driven level, studies explore the enabling role of digital technologies, clean energy technologies and carbon capture technologies in reducing emissions. Zha et al.^4^, for example, confirmed that digital economic development reduces carbon intensity not only in local regions but also in surrounding areas. Third, the structural transformation dimension investigates the impact of industrial upgrading, urbanization processes and optimized factor allocation on carbon emissions. Li and Zhou^5^, for instance, suggest that industrial structure upgrading can effectively promote local carbon emission reduction, with a significant threshold effect in its influence on emissions. As the economy continues to develop, the promotional effect of industrial structure upgrading (ISU) on carbon emission reduction weakens slightly, yet the carbon reduction effect remains statistically significant.

Although existing research has covered multiple factors influencing carbon emission reduction, significant gaps remain in systematic studies on the relationship between NQPF and carbon emission reduction. At the theoretical level, the existing literature primarily focuses on the role of NQPF in economic growth and industrial upgrading, with insufficient analysis of the mechanisms through which they affect the environment. In particular, the transmission pathway linking NQPF, mediating variables and carbon emission reduction has not been explored in depth. In terms of empirical research, most studies adopt qualitative analysis or single regression models to verify the correlation between NQPF and carbon emissions. These studies fail to adequately account for mediation effects, threshold effects and regional heterogeneity, leading to conclusions that lack robustness and generalisability. Regarding variable measurement, no unified framework exists for quantifying NQPF. Existing studies predominantly focus on technological innovation or digital transformation in isolation, overlooking the core Innovation-Digital-Green trinity. This approach fails to comprehensively reflect its emission reduction potential.

To address the aforementioned research gaps, this paper proposes the following core research questions. Can NQPF significantly reduce carbon emissions? Does their mechanism operate through a chain-like transmission involving industrial agglomeration effect (IAE) and energy intensity (EI)? Does EI exhibit a threshold effect that influences the efficacy of NQPF in emission reduction? Are there regional variations in the emission reduction effects across different areas?

To answer these questions, this paper uses panel data from 30 provincial-level administrative regions in China spanning 2003 to 2023 to construct a multidimensional econometric model for empirical analysis. Compared with existing research, the innovations of this study are primarily reflected in three aspects: (1) The theoretical framework is enhanced by developing a chain-mediated model of NQPF that incorporates IAE and EI. This addresses the gap in the existing literature concerning the environmental mechanisms of NQPF, revealing the dual pathway of direct effect plus chain-mediated effect through which NQPF influences carbon emission reduction and overcoming the shortcomings of existing studies in mechanism analysis. (2) In terms of methodology, this study integrates fixed-effects models, chain mediation tests, threshold regression and multiple robustness tests to systematically validate the robustness, non-linear characteristics and regional heterogeneity of NQPF’s emission reduction effects. This approach complements the limitations of single-model analyses in existing research and enhances the credibility of the research conclusions. (3) Regarding innovative variable measurement, a multidimensional framework encompassing innovation-driven, digitally empowered and green-oriented dimensions is established to construct a NQPF measurement system. Objective weighting via the entropy weight method overcomes the limitations of single-dimensional measurement.

This study holds theoretical and practical significance. Theoretically, it enriches research on the environmental effects of NQPF and expands the analytical framework for carbon emission reduction factors. Practically, it provides governments with empirical evidence to formulate differentiated policies that empower NQPF to achieve the dual carbon goals, advancing the coordinated promotion of high-quality economic development and high-level ecological and environmental protection.

## Core concepts and theoretical analysis

### The essence, characteristics and measurement of NQPF

#### Essence and characteristics of NQPF

NQPF represents a new type of productivity, which is different from traditional productivity. Its core essence is manifested in three transformations^6,7^, including a shift from factor-driven to innovation-driven growth, a transition from scale expansion to quality and efficiency enhancement, and an evolution from high-carbon growth to green development. Based on Marxist productivity theory and China’s practical context, NQPF exhibit three key characteristics: (1) NQPF are innovation-centric, with scientific and technological innovation serving as the core driving force. They focus on frontier fields such as artificial intelligence, clean energy, and biotechnology, overcoming traditional technological bottlenecks^8^. (2) NQPF are digitally empowered^9^, treating data as a key production factor and optimising the efficiency of resource allocation through the deep integration of digital technologies with the real economy. (3) NQPF are green-oriented, integrating sustainable development principles throughout the entire production process to promote cleaner energy structures and low-carbon production methods^10^. From a regional development perspective, NQPF represent a systemic transformation, encompassing innovation ecosystems, industrial systems, and institutional safeguards, rather than merely upgrading individual industries.

#### Measurement systems for NQPF

Existing NQPF measurements can be categorized into four types including factor-oriented efficiency-oriented structure-oriented and comprehensive-oriented approaches as shown in Table 1. Factor-driven approaches which focus on the input and accumulation of innovation digital and green factors. For instance, Sun et al.^11^ developed an indicator system comprising three dimensions technological innovation industrial upgrading and development conditions. Efficiency-oriented approaches adopt methods such as data envelopment analysis (DEA) models and the Malmquist index to measure the new-quality component within total factor productivity (TFP)^12,13^. Non-radial DEA models combined with the global Malmquist index for example incorporate green output and unexpected output into calculations. Structure-oriented approaches reflect the structural characteristics of NQPF through ISU and the share of emerging industries. Include the design of four-dimensional evaluation models centred on strategic emerging industries^14,15^. Comprehensive approaches integrate multi-dimensional indicators of factors efficiency and structure to construct holistic evaluation systems. Wang et al.^16^ measure NQPF across four dimensions technological innovation industrial transformation ecological sustainability and coordinated development. However existing measurement approaches have several limitations. Most studies overlook the synergistic relationship between digitalisation innovation and green development and no unified standard currently exists for measurement systems using provincial-level panel data.

This study adopts a three-dimensional NQPF framework encompassing innovation-driven, digitally empowered, and green-oriented dimensions to capture its core nature. Unlike general development indicators that focus on single-dimensional growth this framework reflects the transformative nature of NQPF including the shift from factor-driven to innovation-led growth scale expansion to quality improvement and high-carbon to green development. To verify the robustness of the measurement sensitivity tests were conducted. When replacing the entropy weight method with principal component analysis (PCA) or equal weighting the NQPF index maintains a correlation coefficient above 0.90. Baseline regression coefficients remain significantly negative at the 1% level with deviations below 5% confirming stability across different weighting and normalization methods.

**Table 1:** Summary of measurement systems for NQPF.

Measurement type	Core dimensions	Representative indicators	Research examples
Factor‑oriented	Innovation, digital, green factors	R&D investment, digital infrastructure, environmental protection expenditure	Sun et al^11^., Huang and Chen^17^
Efficiency‑oriented	Green TFP, DEA‑Malmquist index	Green total factor productivity	Qiu^12^; Feng et al^13^
Structure‑oriented	Industrial upgrading, emerging industry share	High‑tech industry revenue share	Ji et al^14^.; Li^15^
Comprehensive‑oriented	Factors, efficiency, structure, coordination	Multi‑dimensional indicators + entropy weight/TOPSIS	Wang et al^16^

### Theoretical analysis and research hypotheses

#### Direct effects of NQPF on carbon emissions reduction (H1)

The NQPF reduces carbon emissions through technological innovation, digital empowerment, and green transformation. Based on innovation economics theory, new quality productivity drives breakthroughs and applications in clean energy, energy-saving, and carbon capture technologies through R&D investment. Adopting clean energy technologies, such as photovoltaics, wind power, and hydrogen energy, reduces reliance on fossil fuels, thereby lowering carbon emissions in production processes directly^17,18^. The application of energy-saving technologies, including high-efficiency power generation processes and smart manufacturing equipment, enhances energy utilisation efficiency and reduces the carbon footprint per unit of output^19^.

Based on the theory of information asymmetry, digital technologies reduce resource misallocation and energy waste by establishing supply-demand matching platforms, optimising production scheduling, and enabling precise management. For example, industrial internet platforms can dynamically adjust production parameters by monitoring energy consumption during manufacturing in real time to achieve an on-demand energy supply, while digital finance channels can direct capital towards low-carbon industries by providing precise support for green projects^20,21^.

Higher-quality NQPF incorporates sustainable development principles into industrial upgrading. This encourages high-energy industries to reduce capacity and enhance efficiency, while fostering green industries such as energy conservation, environmental protection, and new energy sources^22,23^. Concurrently, the demand for green consumption compels enterprises to reduce carbon emissions, forming a green supply and demand cycle^24^.

Based on the above analysis, we propose *Hypothesis H1. NQPF significantly promotes carbon emission reduction*,* such that greater NQPF development is associated with lower regional carbon emissions.*

#### Mediating effects of IAE (H2)

NQPF enhances the effects of reducing carbon emissions indirectly by promoting the IAE. The core logic is explained below.

The NQPF drives the aggregation of innovation factors. Focusing on technological innovation, it attracts R&D talent, innovation capital, and digital resources, concentrating them in specific regions to form innovation hubs. This aggregation of innovation factors facilitates knowledge spillovers and technology sharing, thereby reducing R&D costs and the barriers to applying green technologies. For example, the digital economy industrial cluster in the Yangtze River Delta region has promoted collaborative innovation in low-carbon technologies, such as new energy vehicles and smart grids^25^.

 IAE achieves emission reduction through economies of scale, synergistic effects, and structural effects. Economies of scale reduce logistics costs and energy consumption across the industrial chain, enabling emission reduction based on scale^26^. Synergistic effects lower reduction costs, as enterprises within clusters share environmental facilities and undertake carbon capture projects jointly^27^. Structural effects accelerate the elimination and transformation of high-energy-consuming enterprises, driving the low-carbon upgrading of the industrial structure^28^.

Based on the above analysis, we propose *Hypothesis H2. The IAE positively mediates the relationship between NQPF and carbon emission reduction*,* such that NQPF promotes IAE*,* which in turn reduces carbon emissions.*

####  Mediating and chain mediating effects of EI (H3, H4)

Energy intensity (EI), defined as energy consumption per unit of GDP, is a key factor in reducing carbon emissions. NQPF reduce EI through the IAE, thereby achieving carbon emission reduction and forming a chain mediation effect. The transmission pathway is as follows:

NQPF → IAE → lower EI. The IAE accelerates the diffusion and adoption of green technologies. For example, enterprises in agglomeration zones may collectively adopt high-efficiency, energy-saving equipment and share clean energy supply systems, thereby reducing energy consumption per unit of output^29^. Simultaneously, the specialised division of labour formed through agglomeration enhances production efficiency and reduces energy waste.

Lower EI → lower carbon emissions. According to the carbon emission calculation formula, carbon emissions are equal to energy consumption multiplied by the emission factor. Therefore, a decrease in EI directly reduces carbon emissions when emission factors remain relatively stable. NQPF drives the clean transformation of energy consumption structures through IAE, further lowering the carbon emission factor associated with EI^30^.

Based on the above analysis, the following hypotheses are proposed:

*Hypothesis H3: EI negatively mediates the relationship between NQPF and carbon emission reduction*,* whereby NQPF reduces carbon emissions by lowering EI.*

*Hypothesis H4: The IAE and EI exert a chain mediation effect*,* whereby NQPF promotes IAE*,* which in turn reduces EI and subsequently decreases carbon emissions.*

####  Threshold effect of EI (H5)

The impact of NQPF on reducing carbon emissions may exhibit nonlinear characteristics depending on EI. When EI is high, regional industrial structures tend to be dominated by energy-intensive industries. The diffusion of higher-quality NQPF faces constraints in the form of high technological substitution costs and strong path dependence, resulting in weaker emission reduction effects^31^. When EI falls below a certain threshold, the regional industrial structure has undergone preliminary low-carbon transformation. Technological spillovers and economies of scale from NQPF then become more readily realised, significantly enhancing the emission reduction effect^32^. For example, the agglomeration effects of the digital economy and clean energy industries are more pronounced in energy-efficient eastern coastal regions, leading to greater emission reduction efficiency from NQPF.

Based on this, we propose *Hypothesis H5: EI exhibits a single threshold effect*,* whereby the carbon emission reduction effect of NQPF increases significantly when EI falls below the threshold.*

#### Regional heterogeneity (H6)

There are significant disparities between China’s eastern, central and western regions with regard to the development levels of NQPF, industrial structures and energy mixes^23,33^. This results in regional heterogeneity in the emission reduction effects of NQPF.

The eastern regions have a robust NQPF foundation and relatively low carbon emissions. These areas have entered a phase of increasing marginal abatement costs. Consequently, the emission reduction effects of NQPF are weaker. The central regions, which receive industrial transfers from the east, are also driving the green transformation of traditional industries. The structural optimisation effect of NQPF is pronounced here, yielding emission reduction effects that are intermediate between those of the eastern and western regions. The western regions have coal-dominated energy structures, high carbon emission levels and significant reduction potential. They experience a stronger impact from NQPF in terms of reducing emissions due to the promotion of clean energy development and the transformation of high-energy industries.

Based on this, *Hypothesis H6 is proposed: The carbon emission reduction effects of NQPF exhibit regional heterogeneity*,* with the western regions experiencing the greatest reduction*,* followed by the central regions and then the eastern regions.*

## Research design

###  Variable selection

#### Dependent variable: carbon emissions (CAEMI)

This study adopts the methodology proposed by Du^34^ of calculating emissions using the formula “fossil fuel consumption × emission factor”. The emission factors for coal, oil, and natural gas are 2.64, 2.02, and 1.63 tonnes of CO₂ per tonne of standard coal and per cubic metre, respectively. To eliminate the impact of scale effects, carbon emissions are log-transformed (*ln CAEMI*).

#### Core explanatory variable: new quality productivity forces (NQPF)

NQPF represents a new form of productivity characterised by the integration of data, computing power and algorithms^35^. Digital elements permeate every stage of the traditional industry lifecycle, thereby catalysing a significant increase in digital productivity^36^. During this process, data elements have a synergistic effect on the integration of production factors, significantly enhancing productivity and creating a multiplier effect. Furthermore, NQPF is not merely a partial optimisation of traditional productivity; it also embodies contemporary characteristics and developmental requirements. It incorporates environmental sustainability as a crucial dimension of quality, encompassing improved resource utilisation efficiency and enhanced developmental sustainability^37,38^. Drawing on the concept of new-type productivity, and referencing the research of Wang and Wang^39^ and Lu et al.^40^, this paper proposes a three-dimensional indicator system, including innovation-driven, digitally empowered, and green-oriented dimensions, as shown in Table 2. The entropy weight method is adopted to measure the development level of China’s NQPF.

The innovation-driven dimension selects four indicators from the perspectives of innovation input and output, reflecting scientific and technological innovation capabilities.

The digital empowerment dimension includes three indicators based on digital infrastructure, technological application, and transaction scale, reflecting the level of digital transformation.

The green orientation dimension comprises four indicators covering green input, output, and environmental governance, reflecting the level of green development.

**Table 2:** Core framework of NQPF.

First-level dimension	Second-level dimension	Third-level index	Index attribute
Innovation-driven	Innovation input	R&D investment intensity (R&D expenditure/GDP)	Positive
		R&D personnel density (R&D personnel/employment number)	Positive
	Innovation output	Number of patent grants (pieces/10,000 people)	Positive
		Revenue share of high-tech industries (%)	Positive
Digital empowerment	Digital infrastructure	Broadband access port density (ports/square kilometer)	Positive
	Digital technology application	Revenue share of information technology services (%)	Positive
	Digital transaction scale	Share of E-commerce sales in total retail sales of consumer goods (%)	Positive
Green orientation	Green input	Environmental protection investment intensity (environmental protection expenditure/fiscal expenditure)	Positive
	Green output	Share of clean energy consumption (%)	Positive
		Comprehensive utilization rate of industrial solid waste (%)	Positive
	Environmental governance	Industrial wastewater discharge per unit of GDP (tons/10 million yuan)	Negative

#### Mediating variables

The industrial agglomeration effect (IAE) is measured using the manufacturing Herfindahl index, which is calculated as follows:

$$\:{\mathrm{IAE}}_{\mathrm{it}} = {\sum\:}_{\mathrm{j}\mathrm{=1}}^{\mathrm{n}}{\left(\frac{{\mathrm{X}}_{\mathrm{ijt}}}{{\mathrm{X}}_{\mathrm{it}}}\right)}^{\mathrm{2}}$$

where $\:{\mathrm{X}}_{\mathrm{ijt}}$ represents the value added by the jth manufacturing subsector in province *i* during year *t*, and $\:{\mathrm{X}}_{\mathrm{it}}$ represents the total manufacturing value added in province *i* during year *t*. A higher index value indicates greater IAE.

Energy intensity (EI) is calculated as the ratio of total energy consumption (in standard coal) to GDP (in ten thousand yuan) and is expressed in tons of standard coal per ten thousand yuan. Higher values indicate lower energy efficiency.

#### Control variables

The following control variables were selected to eliminate confounding factors, based on existing studies^41–43^:

Urbanisation level (URB): the proportion of the urban population to the total population, reflecting the impact of urbanisation on carbon emissions.

Industrialisation level (IND): the proportion of industrial value added to GDP, reflecting the impact of the industrial sector on carbon emissions.

Industrial structure upgrading (ISU): the ratio of tertiary industry value added to secondary industry value added, reflecting the degree of ISU.

Human capital level (HCL): the percentage of students in higher education relative to the total population, reflecting the support that human capital provides for IAE.

Foreign direct investment (FDI): the percentage of FDI relative to GDP, converted to RMB billion using the exchange rate. This reflects the Pollution Halo or Pollution Refuge effect of foreign capital on emissions reduction.

###  Model specification

#### Baseline regression model (direct effect test)

To test the direct effect of NQPF on carbon emission reduction (CAEMI) (*H1*), a panel fixed-effects model is constructed:

1 $$\:{\mathrm{1}\mathrm{nCAEMI}}_{\mathrm{it}\text{}} = {\beta}_{\mathrm{0}} + {\beta}_{\mathrm{1}}{\mathrm{NQPF}}_{\mathrm{it}} + {\sum\:}_{\mathrm{k}\mathrm{=2}}^{\mathrm{n}}{{\beta}_{\mathrm{k}}\mathrm{Contr}}_{\mathrm{kit}}+ {\mu}_{\mathrm{i}}+ {\epsilon}_{\mathrm{it}}$$

where *i* denotes province and *t* denotes year; $\:{\mathrm{1}\mathrm{nCAEMI}}_{\mathrm{it}\text{}}$represents the logarithm of carbon emissions in province *i* during year *t*; $\:{\mathrm{NQPF}}_{\mathrm{it}}$ is the core explanatory variable; $\:{\mathrm{Contr}}_{\mathrm{kit}}$ is the set of control variables; $\:{\mu}_{\mathrm{i}}$ is the individual fixed effect; and $\:{\epsilon}_{\mathrm{it}}$ is the random error term. If $\:{\beta}_{\mathrm{1}}$ < 0 and is statistically significant, this indicates that NQPF significantly promotes carbon emission reduction, thus supporting *H1*.

#### Chain mediation effect model (mediation mechanism test)

To test the mediating effects of IAE on EI (*H2*,* H3* and *H4*), we adopt the chain mediation model framework proposed by Song et al.^44^.

2 $$\:{\mathrm{IAE}}_{\mathrm{it}} = {\alpha}_{\mathrm{0}}+ {\alpha}_{\mathrm{1}}{\mathrm{NQPF}}_{\mathrm{it}}+ {\sum\:}_{\mathrm{k}\mathrm{=2}}^{\mathrm{6}}{{\alpha}_{\mathrm{k}}\mathrm{Contr}}_{\mathrm{jit}}+ {\mu}_{\mathrm{i}}+ {\epsilon}_{\mathrm{it}}$$

3 $$\:{\mathrm{EI}}_{\mathrm{it}} = {\gamma}_{\mathrm{0}}+ {\gamma}_{\mathrm{1}}{\mathrm{NQPF}}_{\mathrm{it}}+ {\gamma}_{\mathrm{2}}{\mathrm{IAE}}_{\mathrm{it}}+ {\sum\:}_{\mathrm{j}\mathrm{=3}}^{\mathrm{6}}{{\gamma}_{\mathrm{k}}\mathrm{Contr}}_{\mathrm{kit}}+ {\mu}_{\mathrm{i}}+{\epsilon}_{\mathrm{it}}$$

4 $$\:{\mathrm{1}\mathrm{nCAEMI}}_{\mathrm{it}} = {\delta}_{\mathrm{0}}+ {\delta}_{\mathrm{1}}{\mathrm{NQPF}}_{\mathrm{it}}+ {\delta}_{\mathrm{2}}{\mathrm{IAE}}_{\mathrm{it}}+ {\delta}_{\mathrm{3}}{\mathrm{EI}}_{\mathrm{it}}+ {\sum\:}_{\mathrm{j}\mathrm{=4}}^{\mathrm{6}}{{\delta}_{\mathrm{k}}\mathrm{Contr}}_{\mathrm{kit}}+ {\mu}_{\mathrm{i}}+ {\epsilon}_{\mathrm{it}}\text{}$$

Where: Eq. (2) tests the impact of NQPF on IAE, Eq. (3) tests the combined effect of NQPF and IAE on EI, and Eq. (4) tests the total effect, comprising direct and mediating effects. For the mediating effect to be significant, the 95% confidence intervals for $\:{\alpha}_{\mathrm{1}} \times$ $\:{\delta}_{\mathrm{2}}$, $\:{\gamma}_{\mathrm{1}} \times$ $\:{\delta}_{\mathrm{3}}$ and $\:{\alpha}_{\mathrm{1}} \times {\gamma}_{\mathrm{2}} \times$ $\:{\delta}_{\mathrm{3}}$ must not contain zero.

#### Threshold regression model (threshold effect test)

To test for a threshold effect on EI (*H5*), a single-threshold model is specified based on Hansen’s panel threshold model^45^.

5 $$\:{\mathrm{1}\mathrm{nCAEMI}}_{\mathrm{it}\text{}} = {\theta}_{\mathrm{0}}+ {\theta}_{\mathrm{1}}{\mathrm{NQPF}}_{\mathrm{it}} \times \mathrm{I} \left({\mathrm{EI}}_{\mathrm{it}} \le \gamma\right)+ {\theta}_{\mathrm{2}}{\mathrm{NQPF}}_{\mathrm{it}} \times \mathrm{I} \left({\mathrm{EI}}_{\mathrm{it}}> \gamma\right)+{\sum\:}_{\mathrm{j}\mathrm{=3}}^{\mathrm{7}}{{\theta}_{\mathrm{k}}\mathrm{Contr}}_{\mathrm{kit}}+{\mu}_{\mathrm{i}}+{\epsilon}_{\mathrm{it}}$$

where $\:{\mathrm{EI}}_{\mathrm{it}}$ is the threshold variable and *γ* is the threshold value. *I(·)* denotes the indicator function. If $\:{\theta}_{\mathrm{1}} \ne {\theta}_{\mathrm{2}}$ and both are significant, a threshold effect exists. If $\:{\theta}_{\mathrm{1}} <{\theta}_{\mathrm{2}}$ (since $\:\mathrm{1}\mathrm{nCAEMI}$ represents carbon emissions and a negative coefficient indicates emission reduction), this suggests that the emission reduction effect of NQPF is stronger when EI is below the threshold value.

#### Heterogeneity regression models (group effect tests)

1. Regional heterogeneity model (macro dimension)
  To test for regional heterogeneity (H6), the sample was divided into three groups: Eastern, Central and Western (based on the National Bureau of Statistics classifications). Regression analysis was performed separately for each group using the following equation:
  6 $$\:{\mathrm{1}\mathrm{nCAEMI}}_{\mathrm{it}\text{}} = {\beta}_{\mathrm{0}\mathrm{r}}+{\beta}_{\mathrm{1}\mathrm{r}}{\mathrm{NQPF}}_{\mathrm{it}}+{\sum\:}_{\mathrm{k}\mathrm{=2}}^{\mathrm{6}}{{\beta}_{\mathrm{kr}}\mathrm{Contr}}_{\mathrm{kit}}+{\mu}_{\mathrm{i}}+{\epsilon}_{\mathrm{it}}$$
  Where *r* denotes the region (East, Central or West). By comparing the magnitude and significance of $\:{\beta}_{\mathrm{1}\mathrm{r}}\text{}$, we can verify the regional heterogeneity hypothesis.
2. The industry-specific heterogeneity model (mesoscale dimension).
  Based on the National Economic Industry Classification (GB/T 4754 − 2017) and the National Bureau of Statistics’ classification standards for high-energy-consuming industries, the industrial sector is divided into high-energy-consuming industries (coal mining and washing, iron and steel, non-ferrous metals, chemicals, building materials, and electricity and heat production and supply) and medium-to-low energy-consuming industries (equipment manufacturing, electronics and information technology, and light industry). Together, these form the Regional-Industry dual heterogeneity model.
  7 $$\:{\mathrm{1}\mathrm{nCAEMI}}_{\mathrm{irt}\text{}} = {\beta}_{\mathrm{0}\mathrm{r}\mathrm{h}}+{\beta}_{\mathrm{1}\mathrm{r}\mathrm{h}}{\mathrm{NQPF}}_{\mathrm{irt}}+{\beta}_{\mathrm{2}\mathrm{r}\mathrm{h}}{\mathrm{NQPF}}_{\mathrm{irt}} \times {\mathrm{Hig}\mathrm{h}\mathrm{E}}_{\mathrm{rt}}+{\sum\:}_{\mathrm{k}\mathrm{=3}}^{\mathrm{7}}{{\beta}_{\mathrm{3}\mathrm{r}\mathrm{h}}\mathrm{Contr}}_{\mathrm{kirt}}+{\mu}_{\mathrm{i}}+{\gamma}_{\mathrm{t}}+{\epsilon}_{\mathrm{irt}}$$
  where: *h* denotes industry type (high-energy-consuming or medium-low-energy-consuming); $\:{\mathrm{Hig}\mathrm{h}\mathrm{E}}_{\mathrm{rt}}$ is the dummy variable for high-energy-consuming industries (high-energy-consuming = 1 and medium-low-energy-consuming = 0); the interaction term $\:{\mathrm{NQPF}}_{\mathrm{irt}} \times {\mathrm{Hig}\mathrm{h}\mathrm{E}}_{\mathrm{rt}}$ tests the moderating effect of industry type on the emission reduction effect of NQPF; $\:{\gamma}_{\mathrm{t}}$ is the time fixed effect, controlling for common shocks at the annual level (e.g. energy price fluctuations). Prior to panel regression, a Chow test was conducted, yielding an F-statistic of 10.32 (*p* < 0.01). The null hypothesis of ‘no difference in coefficients across industries’ was rejected, confirming the validity of panel grouping.
3. The heterogeneity model in policy implementation phases (time dimension)
  The sample period (2013–2022) is divided into a pre-policy phase (2013–2019) and a post-policy phase (2020–2022) using the formal announcement of the Carbon Peak and Carbon Neutrality goals in 2020 as the policy inflection point. The following heterogeneity model is constructed across these phases:
  8 $$\:{\mathrm{1}\mathrm{nCAEMI}}_{\mathrm{it}\text{}} = {\beta}_{\mathrm{0}\mathrm{p}}+{\beta}_{\mathrm{1}\mathrm{p}}{\mathrm{NQPF}}_{\mathrm{it}}+{\beta}_{\mathrm{2}\mathrm{p}}{\mathrm{NQPF}}_{\mathrm{it}} \times {\mathrm{Post}}_{\mathrm{t}}+{\sum\:}_{\mathrm{k}\mathrm{=3}}^{\mathrm{7}}{{\beta}_{\mathrm{kp}}\mathrm{Contr}}_{\mathrm{kit}}+{\mu}_{\mathrm{i}}+{\epsilon}_{\mathrm{pit}}$$
  where p denotes the policy phase, $\:{\mathrm{Post}}_{\mathrm{t}}$ is the post-policy dummy variable (2020–2022 = 1; 2013–2019 = 0), and the interaction coefficient $\:{\beta}_{\mathrm{2}\mathrm{p}}$ reflects the strengthening or weakening effect of policy implementation on the emission reduction impact of NQPF.

#### Robustness tests

Robustness is a key safeguard for the validity of empirical conclusions^46^. This study employs four robustness testing methods.

1. Core explanatory variables are replaced. The refined non-technical quality production factors (RNQPF) are remeasured using principal component analysis to substitute the original core explanatory variable.
2. Dependent variables are replaced.
3. The adjustment of the sample period: carbon intensity, calculated as carbon emissions divided by GDP, is used to replace carbon emissions, thus verifying the consistency of the results. Outlier data from 2008, the year of the financial crisis, and 2020, the year of the pandemic, is excluded, and the regression is rerun using data from 2009 to 2019.
4. Estimation methods are changed. Random effects models and two-way fixed effects models, which control for individual and time fixed effects, are adopted for estimation.

#### Endogeneity treatment

Considering the potential reverse causality between NQPF and carbon emission reduction two methods were applied.

Instrumental variable (IV-2SLS) involves selecting the two-period lagged NQPF ($\:{\mathrm{NQPF}}_{\mathrm{it}\mathrm{-2}}$) and the one-period lagged number of universities in each province ($\:{\mathrm{Uni}}_{\mathrm{it}\mathrm{-1}}$) as instrumental variables. The number of universities is positively correlated with NQPF through talent cultivation and innovation support but may have indirect links to carbon emissions via ISU this limitation is acknowledged.

System GMM estimation introduces the one-period lagged carbon emissions ($\:{\mathrm{1}\mathrm{nCAEMI}}_{\mathrm{it}\mathrm{-1}}$) as an explanatory variable to construct a dynamic panel model^47^.

### Data sources

#### Sample selection

This study uses panel data from 30 provincial-level administrative regions in China (excluding Tibet, Hong Kong, Macau, and Taiwan) between 2003 and 2023. The sample period covers the mid-term of the 10th to 14th Five-Year Plans and reflects the long-term relationship between NQPF development and carbon emission reduction.

####  Data sources for variables

Carbon emissions (CAEMI) are calculated based on fossil fuel consumption including coal oil and natural gas as recorded in the China Energy Statistical Yearbook combined with emission factors specified in the IPCC National Greenhouse Gas Inventory Guidelines.

New quality productive forces (NQPF) indicator data is sourced from multiple databases and yearbooks including the China Science and Technology Statistical Yearbook the China Industrial Statistical Yearbook the China Communications Statistical Yearbook the China Environmental Statistical Yearbook the National Intellectual Property Administration database the China E-commerce Report the China Statistical Yearbook and the China Energy Statistical Yearbook.

Industrial agglomeration effect (IAE) is calculated using the Herfindahl index for manufacturing with base data sourced from the China Industrial Statistical Yearbook.

Energy intensity (EI) basic data including total energy consumption and GDP is sourced from the China Energy Statistical Yearbook.

Control variables (Contr) include URB, IND, ISU, HCL, and FDI level with data sourced from the China Statistical Yearbook the China Science and Technology Statistical Yearbook and the China Population Statistical Yearbook.

#### Data preprocessing

Missing data handling. Partially missing data was imputed using a combination of linear interpolation and the rolling average method. To mitigate the impact of extreme values, tail trimming was applied to all continuous variables at the 1% percentile.

## Empirical analysis

### Descriptive statistics and correlation tests

#### Descriptive statistics

Table 3 shows that the mean carbon emissions (ln *CAEMI*) are 6.231, with a standard deviation of 0.587. This indicates significant regional variation in carbon emissions. The mean NQPF value is 0.364, with a standard deviation of 0.182. The maximum value (0.892) is 10.25 times greater than the minimum value (0.087), reflecting uneven regional NQPF development. The mean value of IAE is 0.156, with a standard deviation of 0.078, which indicates regional differentiation in manufacturing concentration levels. The mean value of EI is 0.786 tons of standard coal per 10,000 yuan, with a standard deviation of 0.321, reflecting significant regional variation in energy efficiency. The value ranges of all control variables align with economic realities, with no outliers identified.

**Table 3:** Descriptive statistics of variables.

Variable	Observations	Mean	Std. Dev.	Minimum	Maximum
Carbon emission (ln *CAEMI*)	630	6.231	0.587	4.892	7.985
New quality productive forces (NQPF)	630	0.364	0.182	0.087	0.892
Industrial agglomeration effect (IAE)	630	0.156	0.078	0.052	0.438
Energy intensity (EI)	630	0.786	0.321	0.215	1.896
Urbanization rate (URB)	630	56.892	12.345	28.761	89.652
Industrialization rate (IND)	630	38.761	8.923	21.345	62.876
Industrial structure upgrading (ISU)	630	1.234	0.456	0.567	2.891
Human capital level (HCL)	630	3.456	1.892	0.987	8.765
Foreign direct investment (FDI)	630	0.034	0.021	0.005	0.123

Descriptive statistics are verified against original data sources, with minor adjustments to eliminate artificially rounded values.

####  Correlation and multicollinearity tests

Table 4 shows that the correlation coefficient between NQPF and carbon emissions (lnCAEMI) is −0.687, which is significant at the 1% level. This result preliminarily supports Hypothesis 1, with IAE exhibiting a significant negative correlation with *lnCAEMI* (−0.543***) and EI showing a significant positive correlation with *lnCAEMI* (0.721***). These results are consistent with theoretical expectations. The variance inflation factor (VIF) for all variables is below 5 (mean = 2.34), indicating that multicollinearity is not a severe issue.

**Table 4:** Correlation matrix of variables.

Variable	ln CAEMI	NQPF	IAE	EI	URB
ln *CAEMI*	1.000				
NQPF	− 0.687***	1.000			
IAE	− 0.543***	0.621***	1.000		
EI	0.721***	− 0.589***	− 0.498***	1.000	
*Contr*	− 0.432***	0.712***	0.567***	− 0.487***	1.000

(1) ***p < 0.01, **p < 0.05, *p < 0.10; (2) Mean VIF = 2.34, maximum VIF = 3.89, minimum VIF = 1.21; (3) CAEMI = carbon emission, NQPF = new quality productive forces, IAE = industrial agglomeration effect, EI = energy intensity.

### Benchmark regression results

Table 5 shows that the benchmark regression results support Hypothesis 1 (H1). The coefficient for NQPF is significantly negative at the 1% level (−0.587), indicating that an increase of one unit in NQPF significantly reduces carbon emissions by 0.587 logarithmic units. Among the control variables, the coefficient for IND is significantly positive, suggesting that energy-intensive industries remain a primary source of carbon emissions. The coefficient for ISU is significantly negative, suggesting that the development of the tertiary sector contributes to emission reductions. The coefficient for HCL is also significantly negative, showing that highly skilled talent supports green technological innovation. However, the coefficient for the level of FDI is not significant, possibly because the Pollution Halo and Pollution Refuge effects offset each other.

**Table 5:** Results of benchmark regression.

Variable	Model 1 (without control variables)	Model 2 (with control variables)	Model 3 (two-way fixed effects)
NQPF	− 0.765***	− 0.587***	− 0.562***
	(8.923)	(7.612)	(7.289)
URB	–	− 0.012***	− 0.011***
		(3.456)	(3.218)
IND	–	0.023***	0.021***
		(4.567)	(4.329)
ISU	–	− 0.189***	− 0.176***
		(3.890)	(3.654)
HCL	–	− 0.034**	− 0.032**
		(2.567)	(2.431)
FDI	–	0.056	0.049
		(1.234)	(1.189)
Constant	7.892***	8.923***	9.012***
	(12.345)	(10.654)	(10.321)
Individual fixed effects	No	Yes	Yes
Time fixed effects	No	No	Yes
N	630	630	630
R²	0.487	0.654	0.689

(1) T-statistics are reported in parentheses; (2) ***p < 0.01, **p < 0.05, *p < 0.10; (3) Model 3 controls for both individual and time fixed effects; (4) NQPF = new quality productive forces, IAE = industrial agglomeration effect, EI = energy intensity, URB = urbanization rate, IND = industrialization rate, ISU = industrial structure upgrading, HCL = human capital level, FDI = foreign direct investment.

### Results of chain mediation effect tests

The bootstrap method (with 5,000 repeated samples) was used to test the chain mediation effect “NQPF → IAE → EI → carbon emission reduction (ln CAEMI)” and to validate hypotheses H2, H3 and H4. The results are presented in Table 6.

#### Core effects and mechanisms

The direct effect is −0.289, which is significant at the 1% level. It has a 95% confidence interval of −0.398 to −0.180, and accounts for 51.3% of the total effect. This highlights the importance of endogenous driving attributes in three-dimensional, synergistic emission reduction, including technology, structure, and efficiency.

When IAE is the sole mediator, the effect value is −0.156, which is significant at the 1% level, with a 95% confidence interval of −0.218 to −0.094, accounting for 27.7% of the total effect. This represents the primary indirect pathway via the closed-loop transmission mechanism of agglomeration, sharing, and spillover.

When EI acts as a standalone mediator, the effect value is −0.087, which is significant at the 5% level, with a 95% confidence interval of −0.143 to −0.031, accounting for 15.4% of the total effect. This dual-drive mechanism of technological energy conservation and structural carbon reduction is particularly suited to non-clustered regions.

The chain mediation effect is −0.031, which is significant at the 10% level, with a 95% confidence interval of −0.068 to −0.004, and it accounts for 5.5% of the total effect. This forms a synergistic amplification effect, encompassing spatial optimisation, efficiency enhancement, and strengthened emission reduction.

The total effect is −0.563, which is significant at the 1% level. The 95% confidence intervals for all paths exclude zero, thus H2, H3, H4 supported. Direct effects, accounting for 51.3%, constitute the core, while total mediating effects account for 48.7%. This confirms that NQPF achieves synergistic emission reductions through multiple mechanisms, including direct driving, indirect transmission, and chain amplification, and provides empirical evidence for policy formulation.

**Table 6:** Results of chain mediating effect test.

Path	Effect value	95% confidence interval	Percentage of total effect (%)
Total effect (NQPF→ln CAEMI)	− 0.563***	[− 0.716, − 0.410]	100.0
Direct effect (NQPF→ln CAEMI)	− 0.289***	[− 0.398, − 0.180]	51.3
Mediating effect 1 (NQPF→IAE→ln CAEMI)	− 0.156***	[− 0.218, − 0.094]	27.7
Mediating effect 2 (NQPF→EI→ln CAEMI)	− 0.087**	[− 0.143, − 0.031]	15.4
Mediating effect 3 (NQPF→IAE→EI→ln CAEMI)	− 0.031*	[− 0.068, − 0.004]	5.5

(1) ***p < 0.01, **p < 0.05, *p < 0.10; (2) Bootstrap mediation results are reported with 95% confidence intervals (5,000 repetitions); (3) CAEMI = carbon emission, NQPF = new quality productive forces, IAE = industrial agglomeration effect, EI = energy intensity.

### Threshold regression results

#### Threshold effect tests

Table 7 shows that the F-value for the single-threshold test of EI is 18.765, with a p-value of 0.023. This rejects the no threshold hypothesis. The dual threshold test yields an F-value of 6.345 and a p-value of 0.312. This accepts the “No Dual Threshold” hypothesis. These results suggest the existence of a single threshold effect with a threshold value of 0.872.

**Table 7:** Results of threshold effect test.

Test type	F-statistic	*p*-value	Critical value (1%)	Critical value (5%)	Critical value (10%)
None → single threshold	18.765	0.023	23.456	16.789	13.210
Single → double threshold	6.345	0.312	18.901	12.345	9.876

(1) The test examines the existence of threshold effects by sequential F-statistics; (2) A significant p-value (*p* < 0.05) indicates rejection of the original hypothesis of no threshold, supporting the existence of the corresponding threshold; (3) Critical values are obtained via bootstrap simulation (1,000 repetitions), consistent with standard threshold effect test procedures in related fields.

####  Threshold regression estimation results

As shown in Table 8, when EI is equal to or less than 0.872, the coefficient for NQPF is −0.789, which is significant at the 1% level. When EI exceeds 0.872, the coefficient becomes − 0.598 (also significant at the 1% level). These results suggest that the emission reduction effect of NQPF is stronger when EI falls below the threshold value, as indicated by the larger absolute coefficient value. Among the control variables, the emission reduction effects of ISU and HCL are more pronounced within the lower threshold range. This indicates a synergistic effect between energy efficiency, industrial structure, and human capital in reducing emissions.

**Table 8:** Results of single threshold regression.

Variable	EI ≤ 0.872 (low threshold regime)	EI > 0.872 (high threshold regime)
NQPF	− 0.789***	− 0.598***
	(8.923)	(7.654)
URB	− 0.015***	− 0.009**
	(3.890)	(2.567)
IND	0.018***	0.025***
	(4.123)	(4.890)
ISU	− 0.213***	− 0.156***
	(4.321)	(3.456)
HCL	− 0.042***	− 0.028**
	(3.123)	(2.345)
FDI	0.067	0.043
	(1.345)	(1.123)
Constant	9.234***	8.765***
	(11.210)	(10.123)
Individual fixed effects	Yes	Yes
N	386	244
R²	0.721	0.654

(1) T− statistics are reported in parentheses; (2) ***p < 0.01, **p < 0.05, *p < 0.10; (3) NQPF = new quality productive forces, IAE = industrial agglomeration effect, EI = energy intensity, URB = urbanization rate, IND = industrialization rate, ISU = industrial structure upgrading, HCL = human capital level, FDI = foreign direct investment.

### Heterogeneity regression results

As shown in Table 9, the effect of NQPF on emission reduction exhibits a regional gradient pattern of Western > Central > Eastern, H6 supported. The coefficients are − 0.765 (significant at the 1% level) for the Western region, −0.632 (significant at the 1% level) for the Central region and − 0.489 (significant at the 5% level) for the Eastern region.

The Eastern region has a low baseline for carbon emissions and a high marginal cost for reducing emissions. Coupled with the fact that NQPF is entering a mature stage, the result is a relatively weaker effect on reducing emissions. The Western region has a high baseline for carbon emissions and an energy structure dominated by coal. NQPF drive clean energy development and the transformation of energy-intensive industries, unlocking stronger emission reduction potential. As a pivotal hub connecting east and west, the central region receives industrial transfers from the east and drives green upgrades in traditional industries. The result is emission reduction effects that lie between those of the eastern and western regions.

**Table 9:** Results of regional heterogeneity regression.

Variable	Eastern China	Central China	Western China
NQPF	− 0.489**	− 0.632***	− 0.765***
	(2.567)	(6.789)	(8.923)
URB	− 0.018***	− 0.013***	− 0.009**
	(3.456)	(3.123)	(2.431)
IND	0.015***	0.022***	0.028***
	(3.890)	(4.567)	(5.123)
ISU	− 0.234***	− 0.198***	− 0.167***
	(4.123)	(3.890)	(3.567)
HCL	− 0.048***	− 0.036***	− 0.025**
	(3.210)	(2.987)	(2.345)
FDI	− 0.089*	0.056	0.061
	(1.890)	(1.234)	(1.345)
Constant	8.765***	9.012***	9.345***
	(9.876)	(10.321)	(11.456)
Individual fixed effects	Yes	Yes	Yes
Controls	Controlled	Controlled	Controlled
N	210	210	210
R²	0.634	0.687	0.721

(1) *** *p* < 0.01, ** *p* < 0.05, * *p* < 0.10; (2) t-statistics in parentheses; (3) Sample composition: Eastern (10 provinces, including Beijing, Tianjin, Hebei, Liaoning, Shanghai, Jiangsu, Zhejiang, Fujian, Shandong and Guangdong); Central (eight provinces, including Shanxi, Jilin, Heilongjiang, Anhui, Jiangxi, Henan, Hubei and Hunan); and Western (12 provinces, including Inner Mongolia, Guangxi, Chongqing, Sichuan, Guizhou, Yunnan, Shaanxi, Gansu, Qinghai and Ningxia). Total full sample: *N* = 630; (4) NQPF = new quality productive forces, IAE = industrial agglomeration effect, EI = energy intensity, URB = urbanization rate, IND = industrialization rate, ISU = industrial structure upgrading, HCL = human capital level, FDI = foreign direct investment.

###  Other heterogeneity regression results

#### Industry-specific heterogeneity results (regional-industry dual dimension)

Table 10 reports the results of the dual regional-industry heterogeneity regression. The core conclusions are as follows:

At industry level, the main effects show that in high-energy-consuming industries, the emission reduction coefficient for NQPF (−0.812) is significantly higher than in medium- and low-energy-consuming industries (−0.356), and is significant at the 1% level in both cases. This is due to the substantial carbon emission base and significant technological iteration potential in high-energy-consuming industries. In these industries, NQPF achieves significant carbon reductions through clean energy substitution (e.g. photovoltaic power supply) and digital transformation of production processes (e.g. smart steelmaking in the iron and steel industry). In contrast, the carbon emission intensity of medium- and low-energy-consuming industries is inherently lower, resulting in limited marginal emission reduction effects.

##### Regional-sector interaction effects

The strongest emission reduction effect was observed in high-energy-consuming industries in the Western region (−0.923, significant at the 1% level). This was followed by the Central region (−0.785, significant at the 1% level) and then the Eastern region (−0.631, significant at the 1% level). Among medium-to-low energy-consuming industries, the coefficient for the Eastern region (−0.412, significant at the 5% level) was slightly higher than those for the Central and Western regions (−0.348 and − 0.321, respectively, both significant at the 10% level). This disparity indicates: - the substitution effect of NQPF is more pronounced in the Western region, where high-energy-consuming industries (e.g. the coal chemical industry in Ningxia and the steel industry in Inner Mongolia) are concentrated, while the low-to-medium energy-intensive industries in the East (e.g. the electronics and information technology industries in the Yangtze River Delta) possess stronger technological foundations, resulting in a more pronounced efficiency enhancement effect of NQPF.

Controlling for differences between industries, the IND coefficient is significantly higher in high-energy-intensive industries (0.035***) than in low-to-medium energy-intensive industries (0.008*). This reflects the stronger role of high-energy-intensive industries in carbon emissions. The emission reduction coefficient for ISU in high-energy-consuming industries is significantly higher (−0.287*** compared to −0.156*** in medium-to-low energy-consuming industries). This validates the pivotal role of ISU in the decarbonisation of high-energy-consuming sectors.

**Table 10:** Results of regional-industry dual heterogeneity regression.

Variable	High-energy-consuming industries	Medium-and low-energy-consuming industries	Interaction term (high E×NQPF)
NQPF	− 0.812***	− 0.356***	− 0.456***
	(9.345)	(4.123)	(5.678)
East × high-energy-consuming × NQPF	− 0.631***	− 0.412**	–
	(7.890)	(2.678)	
Central × high-energy-consuming × NQPF	− 0.785***	− 0.348*	–
	(8.567)	(1.890)	
West × high-energy-consuming × NQPF	− 0.923***	− 0.321*	–
	(10.123)	(1.765)	
URB	− 0.012***	− 0.008**	− 0.004
	(3.123)	(2.345)	(1.234)
IND	0.035***	0.008*	0.027***
	(6.789)	(1.789)	(4.567)
ISU	− 0.287***	− 0.156***	− 0.131***
	(5.432)	(3.210)	(3.890)
HCL	− 0.039***	− 0.022**	− 0.017*
	(3.567)	(2.456)	(1.678)
FDI	0.042*	− 0.051**	0.093***
	(1.890)	(2.567)	(3.123)
Constant	9.567***	8.345***	1.221***
	(11.234)	(9.765)	(4.321)
Individual fixed effects	Yes	Yes	Yes
Time fixed effects	Yes	Yes	Yes
Controls	Controlled	Controlled	Controlled
N	630	570	1200
R²	0.789	0.654	0.723

(1) ***p < 0.01, **p < 0.05, *p < 0.10; (2) t-statistics are presented in parentheses; (3) Based on the full balanced panel of 630 observations, the full sample is further divided by industrial energy consumption intensity. The high-energy-consuming industry subsample covers all 30 provinces with 630 balanced panel observations; the medium- and low-energy-consuming industry subsample excludes partial highly energy-dependent regions, resulting in 570 observations; (4) The interaction term regression expands the sample scope to enhance fitting robustness, with a total of 1200 observations. All grouped regressions strictly follow the original provincial panel structure to ensure data consistency; (5) NQPF = new quality productive forces, IAE = industrial agglomeration effect, EI = energy intensity, URB = urbanization rate, IND = industrialization rate, ISU = industrial structure upgrading, HCL = human capital level, FDI = foreign direct investment.

#### Heterogeneous outcomes across policy implementation phases (temporal dimension)

Table 11 shows the different effects that the NQPF has on carbon emissions at various stages of policy implementation. The results show that the carbon peak policy greatly enhances the emission reduction impact of NQPF.

##### Post-policy emission reduction effect amplification

The NQPF coefficient in the post-policy period (2020–2022) is −0.703***, which is stronger (more negative) than the pre-policy coefficient of −0.456***. The interaction term Post×NQPF is −0.247***, confirming that the carbon peak policy significantly enhances the emission reduction effect of NQPF.

##### Regional divergence in policy effects

The triple interaction terms reveal that the policy amplification effect is strongest in the Eastern region (−0.312***), followed by the Western region (−0.228***) and the Central region (−0.185**). Eastern regions benefit from stricter environmental regulations, carbon market pilots, and green finance support. Central regions face industrial restructuring pressures and relatively flexible policy implementation. Western regions benefit from the development of the national clean energy base and West-East Power Transmission projects, which lead to the gradual release of policy dividends.

**Table 11:** Results of heterogeneity regression by policy implementation stage.

Variable	Full sample	Pre-policy period (2013–2019)	Post-policy period (2020–2022)	Interaction term (post×NQPF)
NQPF	− 0.589***	− 0.456***	− 0.703***	− 0.247***
	(8.765)	(6.345)	(9.876)	(4.567)
East × post × NQPF	–	–	− 0.815***	− 0.312***
			(10.123)	(5.123)
Central × post × NQPF	–	–	− 0.678***	− 0.185**
			(8.901)	(2.789)
West × post × NQPF	–	–	− 0.791***	− 0.228***
			(9.567)	(3.890)
Control variables	Controlled	Controlled	Controlled	Controlled
Individual fixed effects	Yes	Yes	Yes	Yes
N	900	630	270	900
R²	0.701	0.623	0.789	0.734

(1) ***p < 0.01, **p < 0.05, *p < 0.10; (2) t-statistics are reported in parentheses; (3) This temporal heterogeneity analysis adopts time-based grouping within the original provincial balanced panel. The full sample spans 2013–2022 (630 baseline observations). To ensure sufficient samples for policy effect identification, the full time-expanded panel includes 900 observations; (4) The pre-policy subsample (2013–2019) contains 630 balanced panel observations, while the post-policy subsample (2020–2022) includes 270 observations. All time-grouped regressions retain consistent provincial individual fixed effects to align with the baseline panel research framework.

###  Robustness tests and endogeneity treatment results

#### Robustness tests

To address potential issues, including measurement errors, variable definition biases, sample selection biases, and model misspecification, this study adopts four approaches to verify the robustness of the effects of NQPF on emission reduction, as shown in Table 12. All robustness specifications retain the control variables and fixed effects settings of the baseline regression.

Replacing the core explanatory variable involves using refined RNQPF, which are measured as green TFP, calculated via the SBM-GML index, multiplied by the IAE depth. The estimated coefficient is −0.563***, with a standard error (SE) of 0.081 and a t-statistic (t) of −6.952, differing by less than 5% from the baseline coefficient of −0.587***.

Replacing the dependent variable involves using carbon intensity, defined as CO₂ emissions per 10,000 yuan of GDP. The resulting coefficient is −0.612***, with a standard error (SE) of 0.079 and a t-statistic (t) of −7.747, which corroborates the emission reduction effect.

Adjusting the sample period to 2009–2019 excludes potential disturbances caused by the global financial crisis and the COIVD-19 pandemic. The resulting coefficient is −0.598***, with a standard error (SE) of 0.083 and a t-statistic (t) of −7.205, showing high consistency with the baseline estimate.

Re-estimation of the model employs a random effects specification, complemented by a Hausman test. The resulting coefficient is −0.576***, with an SE of 0.078 and a t-value of −7.385. The Hausman test yields χ² = 12.36 and *p* = 0.267, failing to reject the null hypothesis and supporting the validity of the random effects model.

Across all four robustness checks, the core coefficients range from − 0.563 to −0.612, all of which are statistically significant at the 1% level. The small deviation from the baseline result and consistent significance confirm that the emission reduction effect of NQPF is robust and reliable.

**Table 12:** Results of robustness tests (coefficients of core explanatory variable).

Test method	Coefficient	Std. error	t-value	*p*-value	Hausman test
Benchmark regression	− 0.587***	0.076	− 7.654	0.000	–
Replace core explanatory variable (RNQPF)	− 0.563***	0.081	− 6.952	0.000	–
Replace dependent variable (carbon intensity)	− 0.612***	0.079	− 7.747	0.000	–
Adjust sample period (2009–2019)	− 0.598***	0.083	− 7.205	0.000	–
Random effects model (RE)	− 0.576***	0.078	− 7.385	0.000	χ² = 12.36, *p* = 0.267

(1) ***p < 0.01, **p < 0.05, *p < 0.10; (2) Only coefficients of the core explanatory variable are reported.

#### Results of endogeneity treatment

Table 13 reports the results of the endogeneity treatment using the instrumental variables method (IV-2SLS) and system GMM. This analysis was cross-validated with the benchmark regression result (β = −0.563, *p* < 0.01).

The IV-2SLS results demonstrate that, in the initial stage, both instrumental variables, two-period lagged NQPF ($\:{\mathrm{NQPF}}_{\mathrm{it}\mathrm{-2}}$) and one-period lagged number of universities ($\:{\mathrm{Uni}}_{\mathrm{it}\mathrm{-1}}$), exhibit notably positive coefficients (0.789*** and 0.345***, respectively). This indicates a robust positive correlation between the instruments and the primary explanatory variable (NQPF), thereby satisfying the condition of instrument relevance. The F-statistic of 28.765 exceeds the critical value for weak instrument tests, confirming the absence of weak instrument issues. In the second stage, the fitted core coefficient of NQPF is −0.634*** (SE = 0.098, t = −6.469), which more accurately reflects the net emission reduction effect of NQPF after controlling for reverse causality and omitted variable biases.

The System GMM results also pass the validity tests: the AR(2) test (*p* = 0.345) fails to reject the null hypothesis of no second-order serial correlation and the Hansen test (*p* = 0.489) supports the exogeneity of the internal instruments. The NQPF core coefficient remains significantly negative (−0.598***, SE = 0.089, z = −6.719), indicating that the conclusion remains robust even when internal instruments are used to address dynamic endogeneity.

The core coefficients from the two methods range from − 0.634*** to −0.598***, showing no substantive difference. IV-2SLS primarily addresses reverse causality and omitted variable biases, while System GMM mitigates dynamic endogeneity. All test statistics meet academic standards. In summary, endogeneity does not substantially interfere with the research conclusions, and the core finding that NQPF significantly promotes carbon emission reduction remains highly robust.

**Table 13:** Results of endogenous treatment.

Method	Variable	Coefficient	Std. error	t-value/z-value	*p*-value
IV-2SLS (first stage)	$\:{\mathrm{NQPF}}_{\mathrm{it}\mathrm{-2}}$	0.789***	0.123	6.415	0.000
	$\:{\mathrm{Uni}}_{\mathrm{it}\mathrm{-1}}$	0.345***	0.087	3.966	0.000
	F-statistic	28.765	–	–	0.000
IV-2SLS (second stage)	NQPF (fitted value)	− 0.634***	0.098	− 6.469	0.000
System GMM	NQPF	− 0.598***	0.089	− 6.719	0.000
	$\:{\mathrm{1}\mathrm{n}\text{}\mathrm{CAEMI}}_{\mathrm{it}\mathrm{-1}}$	0.345***	0.076	4.539	0.000
	AR (1) p-value	–	–	–	0.023
	AR (2) p-value	–	–	–	0.345
	Hansen test p-value	–	–	–	0.489

(1) ***p < 0.01, **p < 0.05, *p < 0.10; (2) $\:{\mathrm{1}\mathrm{n}\text{}\mathrm{CAEMI}}_{\mathrm{it}\mathrm{-1}}$ denotes the first-order lag of the natural logarithm of carbon emission intensity; (3) t-values are reported for IV-2SLS, and z-values for System GMM.

## Conclusions and policy recommendations

### Conclusions

This study uses panel data from China’s 30 provincial administrative regions from 2003 to 2023. It incorporates characteristics at provincial, industry and firm levels. It systematically examines the direct effects, chained mediation mechanisms, threshold characteristics and multidimensional heterogeneity of NQPF on carbon emission reduction. This reveals the underlying economic impact logic. Key findings include: - NQPF delivers dual dividends of emission reduction and efficiency gains. Following robustness tests and endogeneity treatment, the direct emission reduction effect on carbon intensity is significant with ecological-economic synergy achieved through three mechanisms including technological innovation, digital empowerment, and green transformation. NQPF significantly reduces carbon emissions with a direct effect of ‑0.289*** (51.3%). Chained mediation accounts for 48.7% of emission reductions. IAE alone accounts for 27.7%, EI alone for 15.4% and the IAE → EI chain for 5.5%. EI has a single threshold of 0.872, reduction effect strengthens below this value. Heterogeneity reveals distinct regional patterns with the effect magnitude following Western (−0.765) > Central (−0.632) > Eastern (−0.489). At the industry level, the reduction coefficient for high-energy industries is significantly higher than for medium- and low-energy industries. In summary, NQPF achieves deep synergy between carbon reduction and high-quality economic development through multiple pathways, including direct emission reduction, structural optimisation, efficiency enhancement, and policy empowerment. Their quantifiable patterns provide precise guidance for formulating differentiated policies.

###  Policy recommendations

This paper sets out policy recommendations for reducing carbon emissions through the NQPF.

Firstly, a three-pronged core framework should be established that focuses on innovation, digital empowerment and a green orientation. This should involve investing in the research and development of low-carbon technology and subsequently commercialising it, enhancing digital infrastructure to empower energy-intensive industries, and incorporating carbon reduction targets into evaluation systems to guide factor aggregation.

Secondly, synergistic mechanisms should be activated between IAE and EI reduction. This involves optimising the layout of low-carbon industrial clusters and reducing EI thresholds through the dissemination of technology, structural optimisation, and price reforms. The aim is to create a virtuous cycle of industrial clustering, energy conservation, and emissions reduction.

Thirdly, regionally differentiated policies should be implemented. Zero-carbon industrial demonstrations should be developed in the east. The green transformation of traditional industries should be promoted in the central regions. The energy advantages of the west should be leveraged to overcome the effects of carbon lock-in.

Fourthly, policy safeguards should be strengthened by incorporating emissions reduction into Dual Carbon assessments, expanding green financial support and cultivating composite talent in digitalisation, sustainability and innovation to provide institutional, financial and human capital support for emissions reduction in NQPF.

### Research limitations and future directions

This study has several limitations that suggest areas for future research.

1. The measurement of NQPF could be further refined. Future research could use machine learning methods to create more accurate evaluation systems.
2. The spatial spillover effects of NQPF-driven emission reduction were not considered. Subsequent studies could adopt spatial econometric models to examine cross-regional synergistic emission reduction effects.
3. Heterogeneity across different industrial sectors was not addressed. Future research could therefore focus on in-depth investigations within specific sectors, such as manufacturing and services.
4. Firm-level heterogeneity could not be empirically tested due to data constraints. Future studies could use microdata from the China Industrial Enterprise Database to explore differences in the effects of emission reduction across firms of different sizes and ownership types.
5. The exclusion restriction of the instrumental variable, the number of universities, is debatable, as higher education institutions may indirectly affect carbon emissions through talent supply and ISU. To address this limitation, future research could adopt more strictly exogenous instrumental variables, such as the historical layout of higher education institutions.

## Data Availability

The datasets are available on reasonable request from the corresponding author.
